# Pocket Singh: Periodontist of the year

**DOI:** 10.4103/0972-124X.60220

**Published:** 2009

**Authors:** D. Arunachalam

**Affiliations:** *Journal of Indian Society of Periodontology, H 11 A South Avenue, Thiruvanmiyur, Chennai - 600 041, India E-mail: smilesindia@gmail.com*


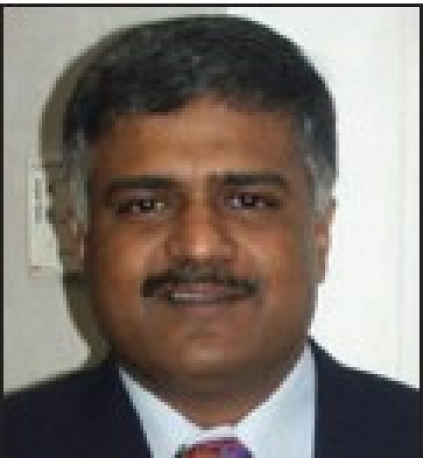


Every general dental practice sees what are in common parlance “scaling cases”; few of them actually are successful in terms of successful recall and review and a minor fraction of them are led on to actual surgical treatment. In most practices, it is the junior doctors who are generally in charge of the “hygiene department,” but brining in a strong periodontal program into their hygiene system will give your practice a significant uniqueness---allow you to practice peridontics!

Here are five ways to develop the periodontal aspect of your prophylactic programs to ensure that periodontal disease is diagnosed, that treatment is accepted and performed, and that payment is received for that treatment.

## ONE CLINIC–ONE VISION

We simply have to increase our productivity by delegating responsibilities to the junior doctors or the hygienists by consistently leaving the prophylaxis procedures to them and sticking to the higher production procedures. This can happen only if this vision is shared with the hygienists and doctors.

While most junior doctors are looking to do ten different things in the practice, guide at least one of them (even if it is the only one you have) into concentrating on a good prophylaxis protocol and get your hands off this hygiene phase. Regular education, good equipments, independence, and confidence building of the associate in front of your patientsall help a lot.

## DIAGNOSE DISEASE

Every patient, currently in your database or new must be thoroughly examined, evaluated, classified, and informed. As periodontists, we are responsible for informing the patient of their current periodontal health status and diagnosing the condition at any stage. Key areas that need to be assessed and recorded include probing pocket depth, bleeding and suppuration upon probing, severity of the bleeding, furcation involvement, recession from the CEJ, mobility, bone loss (recession + pocket depth), attachment loss, and mucogingival health.

Critical to the success of any monitoring program is the rediagnosing at each appointment, giving adequate time to make all these recordings adequate, take radiographs and be able to refer to these radiographs throughout the treatment.

## COMMUNICATE EFFECTIVELY

Effective communication is paramount to enable the patient to understand the need for treatment and to motivate the patient to come for review, recall, and maintenance.

While what is being said is important, how it is being said is equally important. A patient who is lying back in the chair is not in control of the situation. Sitting the patient up will lead to a more effective message getting across.

Taking off your mask or glasses and allowing the patient to see your mouth is more important than you think. At the end of each prophy appointment, a written instruction sheet together with oral hygiene aids of your practice must be handed over to the patient.

Many patients also prefer a private consultation rather than being instructed in the waiting area! And believably case acceptance is enhanced through private consultations.

## USING THE RIGHT TERMS OF TREATMENT

If you are providing a service that is transition of the state of a patient from a diseased state to a healthy state, say so, instead of saying you need a “∼deep cleaning.”

Non-surgical therapy is non-surgical therapy, so let us call it that, schedule adequately and charge the appropriate fee. Perio patients need to be seen and treated differently from routine preventive appointments.

## SET PRODUCTION GOALS FOR THE HYGIENISTS

Your junior doctor or hygienist must necessarily produce at least five times their salary and preblock at least half their appointments for 6 months for period including full mouth series of radiographs or a dental pantomogram.

In conclusion, spend the effort, time, and money to become current with the latest advancement that periodontal therapy can offer and let your junior doctor or hygienist who is competent to handle an effective prophy appointment develop your patient base who will become healthier and stay healthier more than ever.

